# Pretreatment Innate Cell Populations and CD4 T Cells in Blood Are Associated With Response to Immune Checkpoint Blockade in Melanoma Patients

**DOI:** 10.3389/fimmu.2020.00372

**Published:** 2020-03-10

**Authors:** Mehdi R. Pirozyan, Helen M. McGuire, Abdullah Al Emran, Hsin-Yi Tseng, Jessamy C. Tiffen, Jenny H. Lee, Matteo S. Carlino, Alexander M. Menzies, Georgina V. Long, Richard A. Scolyer, Barbara Fazekas de St Groth, Peter Hersey

**Affiliations:** ^1^Melanoma Immunology and Oncology, Centenary Institute, Sydney, NSW, Australia; ^2^Central Clinical School, The University of Sydney, Camperdown, NSW, Australia; ^3^Melanoma Institute Australia, The University of Sydney, Sydney, NSW, Australia; ^4^Ramaciotti Facility for Human Systems Biology, The University of Sydney, Sydney, NSW, Australia; ^5^Discipline of Pathology, Sydney Medical School, The University of Sydney, Sydney, NSW, Australia; ^6^Charles Perkins Centre, The University of Sydney, Sydney, NSW, Australia; ^7^Biomedical Sciences, Faculty of Medicine and Health Sciences, Macquarie University, Sydney, NSW, Australia; ^8^Westmead and Blacktown Hospitals, Sydney, NSW, Australia; ^9^Royal North Shore Hospital, St Leonards, NSW, Australia; ^10^Royal Prince Alfred Hospital, Camperdown, NSW, Australia; ^11^New South Wales Health Pathology, Sydney, NSW, Australia

**Keywords:** melanoma, cancer immunotherapy, NK cells, CyTOF, T cell exhaustion, CD4 T cells, checkpoint inhibition

## Abstract

The development of changes in T cells, referred to as T cell exhaustion, has been suggested as a cause of primary or acquired resistance to immunotherapy by immune checkpoint blockade (ICB). A limited number of studies, largely performed on tumor infiltrating lymphocytes (TILs), has provided evidence in support of this hypothesis, but whether similar changes occur in circulating blood lymphocytes has received little attention. In the present study, a comprehensive analysis of peripheral blood leukocytes from 42 patients taken over the course of treatment with anti-PD-1 was undertaken. The patients included those grouped as responders (who did not progress), primary non-responders (primary resistance) and those with acquired resistance (who initially responded then subsequently progressed). Analysis included surface markers of exhaustion, production of cytokines following *in vitro* stimulation, and assessment of transcription factor levels associated with T cell exhaustion. There were differences in innate cell populations between responders and non-responders at baseline and maintained throughout therapy. Frequencies of total and classical CD14^+^CD16^−^ monocytes were higher and the major subset of NK cells (CD16^hi^CD56^+^) was significantly smaller in the primary resistance group compared with responders. However, differences in peripheral blood expression of exhaustion markers were not evident between the treatment groups. T cell exhaustion markers were expressed in practically all patients and the major observation was an increase in CD39 on CD4 T cells during treatment. The results confirm the association of Eomes transcription factor with T cell exhaustion but levels of expression and the ratio with T-bet over Eomes did not differ between the patient groups. Thus, peripheral blood expression of T cell exhaustion markers does not distinguish between responders and non-responders to anti-PD-1 therapy. CD4 T cell expression of IFNγ also differed in pre-treatment samples, indicating that predictors of response unrelated to exhaustion may be present in peripheral blood. The association of response with innate cell populations and CD4 T cell responses requires further study.

## Introduction

The introduction of immunotherapy based on monoclonal antibodies (mAbs) that block immune checkpoint inhibitors on T cells has been a major advance in treatment of cancers such as melanoma and lung cancer (1, 2). A number of clinical trials over the past few years have established monotherapy with anti-PD-1 or its combination with anti-CTLA-4 as the standard of care in treatment of metastatic melanoma (3–9). Resistance to these treatments is relatively common; between 40 and 60% of patients fail to respond initially, and many that initially respond subsequently progress, such that only 30-40% of patients remain progression-free at 4 years (3, 4, 10). The factors associated with primary and acquired resistance are reviewed elsewhere (5, 6) and include low or absent PD-L1 on melanoma (7) and the degree of T cell infiltration into the tumor. These factors have been used in past studies to classify patients into 4 groups that show different responses to anti-PD-1 therapy (8, 9, 11). Both primary and acquired resistance can be driven by selection of MHC loss variants of the tumor that result in failure of recognition and loss of sensitivity to killing by T cells (12, 13). Study of primary resistant melanoma cells by CRISPR/Cas9 screens revealed loss of antigen presentation as a cause of resistance to immunotherapy (14, 15). Single cell analyses of melanoma tumors prior to anti-PD-1 therapy also identified cellular programs associated with intrinsic resistance driven by CDK4/6 pathways and reversed by inhibitors of CDK4/6 (16). Similar inherent resistance pathways associated with epigenetic changes in the melanoma cells were described in resistance to targeted therapies (17).

In addition to these tumor intrinsic factors, it has been postulated that resistance to immunotherapy may result from decreased T cell function associated with a state referred to as exhaustion, which results from prolonged and repeated antigen stimulation (18, 19). Exhausted T cells are characterized by an altered transcription pattern (20) and by a loss of effector functions such as production of granzyme B and IFNγ, together with expression of multiple inhibitory receptors such as PD-1, LAG-3, TIM-3, and CD39 (21). Exhausted T cells appear to exist in 2 forms; one that is reversible by anti-PD-1 (22) and one that cannot be reversed and is believed to result from more prolonged antigen exposure (23). Recent studies have referred to cells in these 2 states as progenitor and terminally exhausted T cells (24). Single cell RNA-seq analysis on tumor-infiltrating lymphocytes (TILs) from 25 patients revealed that dysfunctional CD8 T cells often formed the majority of proliferating T cells and ranged from 3.6 to 72.1% of the TILs (25). Another single cell analysis of 48 human melanoma samples identified responses to anti-PD-1 to be associated with increased expression of genes coding for TCF7, IL7R, REL, FOXP1, and STAT4 whereas non-response was associated with genes associated with T cell exhaustion, such as CD38, PD-L1, LAG-3, TIM-3, and CTLA-4 (26). Signatures of T cell dysfunction and exclusion (TIDE) in TILs were considered accurate predictors of response to immune checkpoint blockade (ICB) (27).

The exhausted T cell state in cancers has been studied mainly in TILs, but whether this state can be detected in the circulation of cancer patients has received little attention. High dimensional flow cytometry studies on pre-treatment blood samples from 29 patients with metastatic melanoma found that these patients had higher numbers of CD4 FOXP3 T cells and Ki-67 proliferating CD8 T cells compared to normal subjects. Anti-PD-1 antibody treatment resulted in brief increases in Ki-67^+^ CD8^+^ T cells that expressed PD-1, CXCR5, CTLA-4, and 2B4. High Eomes transcription factors were also associated with T cell exhaustion. These changes were seen in 74% of the patients even though clinical responses were seen in only 38% (28), indicating that they are not reliable predictors of response. In another study of 20 melanoma patients treated with anti-PD-1, classical monocytes (CD14^+^CD16^−^) in pre-treatment peripheral blood samples were reported to be strong predictors of response to treatment (29). Similar studies using CyTOF data found that the main differences between responders vs. non-responders to anti-PD-1 were in subsets of NK cells (30, 31).

In the present study, we examined circulating peripheral blood mononuclear cells (PBMCs) for evidence of the exhaustion state by a comprehensive analysis of their expression of markers and transcription factors associated with exhaustion, as well as their production of effector molecules. Longitudinal blood samples were taken from a cohort of patients treated with anti-PD-1. Our results suggest that differences between the responders and non-responders are already evident in pre-treatment samples and undergo very few changes during treatment. They also point to limitations of published studies on PBMCs in understanding the biology of immune responses associated with clinical responses to checkpoint therapy.

## Materials and Methods

### Study Subjects and Samples

**Figure 1:**
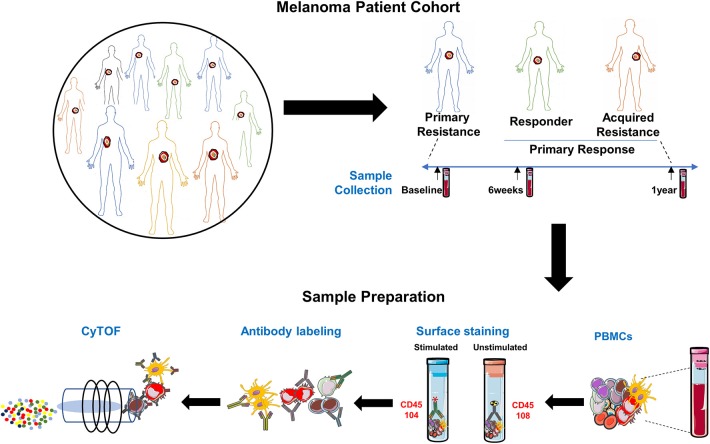
Experimental schema. Experimental setup for processing PBMCs from matched samples before and after anti-PD-1 immunotherapy. Samples (*n* = 97) comprised 36 from melanoma patients prior to beginning anti-PD-1 treatment [Primary Resistance (PR) = 17, Responders (R) = 10 and Acquired Resistance (AR) = 9]; 39 from 6 weeks post-treatment (PR = 19, R = 11, and AR = 9) and 18 from the 1 year time point (PR = 10, R = 2, and AR = 6). PMA/Ionomycin stimulated cells were stained with 104Pd metal tagged anti-CD45 antibody while unstimulated cells from the same sample were stained with 108Pd metal tagged anti-CD45 antibody. Barcoded samples were washed, pooled, and subsequently stained with a panel of 39 antibodies, each conjugated to a different metal isotope, and analyzed by mass cytometry. Cells were nebulized into single-cell droplets, and an elemental mass spectrum was acquired for each. The integrated elemental reporter signals for each cell were then analyzed using a supervised gating strategy (FlowJo). For some analyses, responder and acquired resistance groups were pooled into a primary responder group equivalent to the responder groups in published studies in which progression after 3 month RECIST assessment was not used to subdivide the responder group.

**Figure 2:**
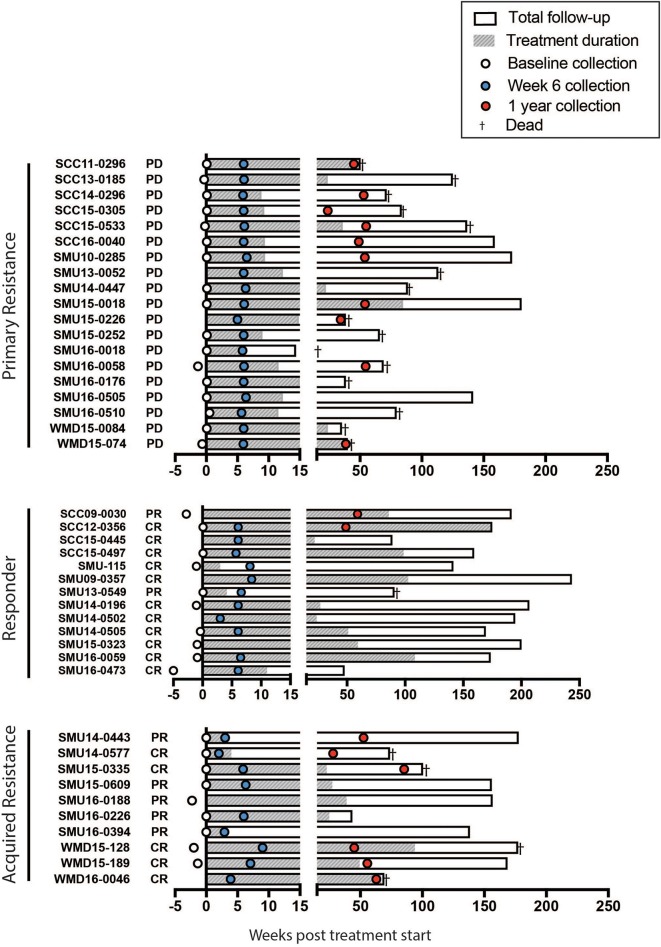
Swimmers plot illustrates treatment duration of patients treated with anti-PD-1 (filled bars), within the study follow up period (open bars) for primary resistance, responders, and acquired resistance groups. Death is illustrated with a cross. Blood samples were obtained at baseline (open circles, range 0-5 weeks before the start of therapy), week 6 (blue filled circles, range 2-9 weeks) and 1 year (red filled circles, range 35-85 weeks) post treatment. CR, complete response; PR, partial response; PD, progressive disease, as assessed using RECIST.

The study schema is shown in Figure 1. Written consent was obtained from all patients at Melanoma Institute Australia, Sydney, Australia, and its affiliated hospitals, and the relevant ethical approval was obtained from Human Research Ethics Committees. Samples were collected under the biospecimen bank protocol No X15-0454 (prev. X11-0289) and HREC/11/RPAH/444. Clinical Data were collected under the MIA Melanoma Research Database protocol: Protocol No X15-0311 (previously X10-0300) and HREC/10/RPAH/530 – “Melanoma Institute Australia: Melanoma Research Database.” A cohort of 42 metastatic melanoma patients treated with anti-PD-1 (Pembrolizumab or Nivolumab) therapy was retrospectively identified (Figure 2, Supplementary Table 1). Baseline bloods were collected before the first administration of anti-PD-1. Based on response evaluation criteria in solid tumors (RECIST) (32), patients were categorized into three groups - progressive disease (primary resistance), objective response (complete or partial) without subsequent progression (responder), and initial objective response followed by subsequent progression (acquired resistance). Baseline samples included 10 responders, 17 non-responders, and 9 acquired resistance. Follow up samples were obtained at ~6 weeks post treatment (range 2-9 weeks) (11 responders, 19 primary resistance, and 9 acquired resistance). However, 1 year follow up samples (range 35-85 weeks) were obtained from only 2 responder, 10 primary resistance, and 6 acquired resistance patients. For some analyses, responder and acquired resistance groups were pooled into a primary responder group equivalent to the responder groups in published studies in which progression after 3 month RECIST assessment was not used to subdivide the responder group.

All whole blood samples were processed to isolate PBMCs by density gradient centrifugation, using Lymphoprep density gradient media or SepMate isolation tubes (Stem Cell Technologies). Single-cell suspensions were then cryopreserved in fetal bovine serum (FBS) supplemented with 10% DMSO (Sigma-Aldrich), using a controlled freezing unit (Cool Cell LX) and then stored in liquid nitrogen for later use. Matched TILs from an acquired resistance patient at the 1 year time point were prepared by manual mincing followed by dissociation into single-cell suspensions using the human Tumor Dissociation Kit and gentleMACSTM Dissociator (Miltenyi Biotec), before cryopreservation as for PBMCs.

### Mass Cytometry Immunophenotyping

For immunophenotyping of PBMCs, a panel of 39 metal-tagged monoclonal antibodies was optimized and employed. Antibody specificities were chosen to provide wide coverage of CD8 T cell markers (27 markers at baseline plus 3 cytokines), with much more limited coverage of markers specific to the NK and myeloid compartments. A detailed list of antibodies and corresponding metal tags is provided in Supplementary Table 2. All antibodies were validated, pre-titered and supplied in per-test amounts by the Ramaciotti Facility Reagent Bank. Reagent bank antibodies were either purchased from Fluidigm (Fluidigm, South San Francisco, CA) in pre-conjugated form or unlabeled antibodies were purchased in a carrier-protein-free format and conjugated at the Ramaciotti Facility with the indicated metal isotope using the MaxPAR conjugation kit (Fluidigm, South San Francisco, CA) according to the manufacturers protocol.

PBMC stimulation, staining and data acquisition by CyTOF were performed as described previously (33). Briefly, PBMC vials were thawed in a 37°C water bath for 2 min before transfer of the suspension into a 15 mL Falcon tube containing 10 mL R10 culture media RPMI-1640 (Gibco, UK) supplemented with 10% FBS and penicillin and streptomycin (Invitrogen, USA) as well as 1:10,000 universal nuclease (Thermo Fisher Waltham, MA). Cell concentration and viability were determined using a TC20 automated cell counter (Bio Rad, USA) and Trypan blue. Subsequently, a maximum of two million cells were left either untreated or stimulated for 3 h at 37°C using 100 nM of phorbol-12-myristate-13 acetate (PMA) (Sigma) and 1 μM of ionomycin in the presence of 10 μg/ml Brefeldin A (Sigma). For samples with insufficient cells, the untreated control was omitted.

Following stimulation, PBMCs were stained using 1.25 μM cisplatin in PBS for 3 min at room temperature and subsequently quenched with R10, in order to discriminate live from dead cells. Barcoding was performed by incubating cells for 30 min with CD45 antibodies conjugated with various metals. Cells were washed twice with FACs buffer (PBS, 0.02% Sodium Azide, 0.5% BSA and 2mM EDTA), differentially CD45-labeled stimulated and unstimulated samples were combined and incubated with antibodies targeting surface antigens for 30 min at 4°C. Following washing with FACS buffer, cells were fixed and permeabilized using eBiosciences FoxP3 buffer kit (San Diego, CA, USA) at 4°C for 45 min and stained with intracellular antibodies for 30 min on ice. Cells were then washed twice and fixed in 4% paraformaldehyde containing DNA intercalator (0.125μM iridium-191/193; Fluidigm) overnight. After multiple washes with FACS buffer and MilliQ water, cells were diluted to 800,000 cells/mL in MilliQ water containing 1:10 diluted EQ beads (Fluidigm) and filtered through a 35-μm nylon mesh. Cells were acquired at a rate of 200–400 cells/second using a CyTOF 2 Helios upgraded mass cytometer (Fluidigm, Toronto, Canada).

Samples were stained and run in 13 batches. When multiple samples from different timepoints were available for a patient, they were included in the same batch. Each batch also included a batch control consisting of one replicate aliquot of PBMC from a healthy donor, thawed and stimulated in parallel with the patient samples.

All .fcs files obtained from the Helios analysis were normalized using the processing function within the CyTOF acquisition software based on the concurrently run EQ four element beads.

### Analysis of Mass Cytometry Data

Data analysis was performed using FlowJo version 10.4 software (FlowJo, LLC, Ashland, OR, USA). Samples were pre-gated on DNA+, live, CD45+ cells, and exported for further analysis. Manual gating was performed independently by two individuals to ensure accuracy. Major populations were gated as indicated in Supplementary Figure 2. Gates were adjusted on the basis of the batch-to-batch variations apparent from the batch control data. Expression of exhaustion markers and cytokines was then examined within CD4 and CD8 T cells and their subpopulations (Figures 4–6, Supplementary Figures 3–6).

The *t*-stochastic neighborhood embedding (*t*-SNE) algorithm was applied using the FlowJo plugin. Files containing live cells with stringent doublet exclusion were down sampled without replacement to 1,000 cells using the FlowJo DownSample plugin. Stimulated and unstimulated samples from all patients plus the 13 batch controls were concatenated separately to yield files containing ~100,000 events suitable for t-SNE analysis. The t-SNE algorithm was run on the 2 concatenated files using the 36 markers shown in Supplementary Table 2 (excluding the cytokines IL-2, TNFα and IFNγ). Marker expression for each patient group was visualized using the tSNE global scaling script (https://github.com/sydneycytometry/tSNEplots) and is shown in Supplementary Figures 7–10.

### Statistical Analysis

Statistical analysis and graphical representation were performed using Graphpad Prism, version 7.0 (GraphPad Software, Inc. USA, La Jolla). Group comparisons were performed as detailed in the figure legends.

## Results

### Patients and Sample Characteristics

A summary of the study participants is presented in Supplementary Table 1. The overall survival of the patient groups is shown in Supplementary Figure 1. As expected the responder patients had a significantly longer survival than non-responders and those with acquired resistance. Blood was collected from a total of 42 patients (responder = 13, primary resistance = 19, and acquired resistance = 10). Details of the sample collection and survival of individual patients in each group are presented in Figure 2.

### Differences in Immune Populations Between the Groups Before and After PD-1 Immunotherapy

The frequencies of the major immune populations were determined using a conventional supervised gating approach as shown in Supplementary Figure 2. Frequencies of B cells, CD4 and CD8 T cells and their naïve and memory subsets, γδT cells, natural killer (NK) cells, and monocytes were calculated as a percentage of live cells for each of the 89 unstimulated samples. There were no significant differences in the frequencies of B cells, CD4 and CD8 T cells or their naïve and memory subsets, or γδT cells between any of the three patient groups, either before or after therapy. When the responder and acquired resistance groups were pooled (as would be the usual practice in studies with shorter followup times when responder status would be determined after the 3 month RECIST assessment), no differences in B cells, CD4 and CD8 T cells or their naïve and memory subsets, or γδT cells were observed.

**Figure 3:**
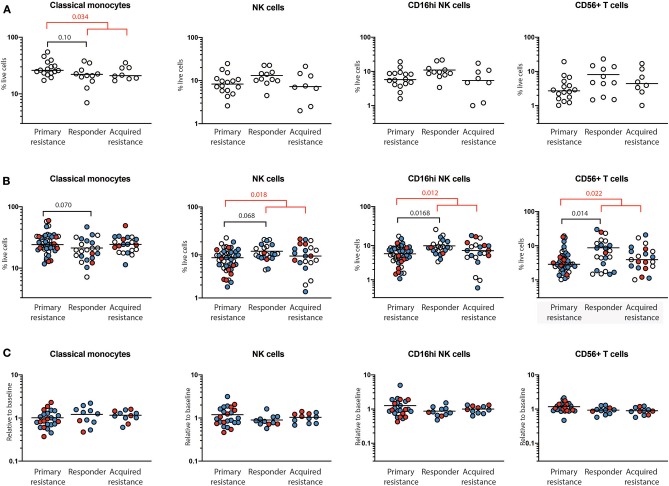
Quantification of immune cells populations using mass cytometry. PBMCs from primary resistance, responder and acquired resistance groups were analyzed for multiple T cell, B cell, NK cell, and myeloid populations, as illustrated in Supplementary Figures 2, 3. Only populations that differed between groups are shown. **(A)** Frequencies of baseline cell populations in each of the groups. Each individual sample is indicated by a circle, with the group mean shown as a bar. **(B)** Frequencies of cell populations from all timepoints in each of the groups. Baseline, open circles; week 6, blue filled circles; 1 year, red filled circles. **(C)** Changes in frequencies within individual subjects over time. Values at 6 weeks, expressed as a proportion of baseline values, are indicated by blue filled circles while values at year 1, expressed as a proportion of baseline values, are indicated by red filled circles. Differences in frequencies of immune cell populations between primary resistance, responder, and acquired resistance groups were assessed using ANOVA followed by Tukey's multiple comparisons test and *p*-values are shown in black. Differences between primary resistance and primary responder (i.e., pooled responder and acquired resistance) groups were assessed using an unpaired *t*-test and *p*-values are indicated in red. Where no *p*-values are indicated, the relevant tests indicated *p* > 0.05.

At baseline, there was a trend toward higher frequencies of total and classical CD14^+^CD16^−^ monocytes in the primary resistance group compared to the responder group (Figure 3A), and this difference was statistically significant when the responder and acquired resistance groups were combined into a single primary responder group and compared with the primary resistance group. When all the timepoints were included in the comparison between responder and primary resistance groups, the increase in monocytes in the primary resistance group was no longer significant (Figure 3B). No difference was observed in MDSCs, identified as CD14^+^HLA-DR^lo^ cells between any of the three patient groups, either before or after therapy. There was a trend to lower NK cells in the primary resistance group at baseline, and the difference reached statistical significance in the all timepoints comparison between the combined primary responder and primary resistance groups. The major subset of NK cells (CD16^hi^CD56^+^) was smaller in the primary resistance group compared with responders in the all timepoints comparison, and also in comparison with the primary responder group (Figure 3B). There was also a trend toward higher CD56 expression within T cells in the responder group, and this reached statistical significance in the all timepoints comparison, both between primary resistance and responder, and primary resistance and primary responder (Figure 3B). Pairwise comparisons revealed no significant correlation between monocyte, NK and CD56^+^ T cell numbers with subjects.

For total NK cells and the CD16^hi^CD56^+^ NK cell subset, the variance of the primary and acquired resistance groups was higher than the responder group (Figure 3B). To test whether this was due to changes over time within a single individual, or to differences that pre-dated therapy and were maintained over time, we expressed the 6-week and 1-year cell subset frequencies as a proportion of baseline for those subjects where baseline values were available (Figure 3C). This analysis revealed that the variances of the changes in NK cell population sizes within individual patients were similar in all 3 groups, and were smaller than the variances of the patient samples within each group. Thus, the frequency of total NK cells and CD16^hi^CD56^+^ NK cells in individual patients was relatively unaffected by therapy. The same was true for CD56^+^ T cells, but not for classical monocytes. These data indicate that patients who show primary resistance to anti-PD1 therapy have a pre-existing deficit in CD56-expressing NK and T cells that remains throughout therapy. In contrast, monocyte frequencies vary as much in individuals over time as between members of each treatment group.

### Expression of Inhibitory Molecules by T Cells According to Clinical Responses

As T cells are the major targets of anti-PD-1 immunotherapy, we compared the expression of five inhibitory markers [PD-1, CD39, TIGIT, TIM-3, and killer cell lectin like receptor G1 (KLRG1)] on CD4 and CD8 T cells and assessed their potential association with clinical responses. Expression of the panel of markers examined on CD8 T cells is shown in Supplementary Figure 3. Some gates were set according to expression on a matched TILS sample, since PD-1, CD39, TIGIT, and TIM-3 are all expressed by a far lower proportion of PBMC than TILS (Supplementary Figure 4). There were no significant differences in the response group frequencies of CD4 or CD8 T cells expressing PD-1, CD39, TIGIT, TIM-3, or KLRG1 when compared at baseline, at 6 weeks or at 1 year (not shown).

**Figure 4:**
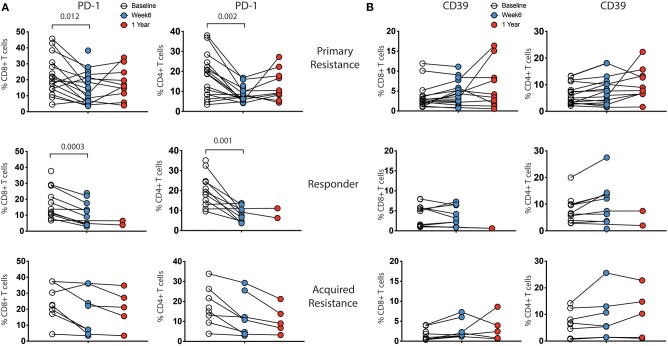
Expression levels of inhibitory receptors on CD4 and CD8 T cells by treatment outcome. PBMCs were analyzed for expression of CD39, PD-1, TIGIT, TIM-3, and KLRG1 on CD4 and CD8 T cells. There were no significant differences in expression between the groups at any timepoint. Expression of **(A)** PD-1 and **(B)** CD39 showed changes within individual subjects over the course of therapy, while TIM-3, TIGIT, and KLRG1 did not (not shown). Baseline, open circles; week 6, blue filled circles; 1 year, red filled circles. Differences in frequencies of immune cell populations between the 3 timepoints were assessed using a repeated measures mixed effects analysis followed by Tukey's multiple comparisons test and *p*-values are indicated.

Longitudinal analysis showed a reduction in expression of PD-1 within both CD8 and CD4 T cells at week 6 post treatment, as expected (Figure 4A). The reduction was of similar magnitude in both responder and primary resistance groups. Interestingly, PD-1 expression rebounded strongly in only 2 of the 7 primary resistance group patients whose 1-year sample was taken after cessation of therapy. There was an increase from baseline to week 6 in the frequency of CD39-expressing CD4 T cells in the responder and primary resistance groups, but this trend did not reach statistical significance.

We analyzed the combination of expression of three inhibitory markers (CD39, PD-1, and TIM-3), using a Boolean gating approach. These markers have been recently shown to predict terminally exhausted T cells in melanoma patients (21). The major CD8 subpopulations consisted of CD39^−^PD-1^−^Tim-3^−^ cells followed by CD39^−^PD-1^+^Tim-3^−^ cells. Overall, we did not see significant differences in any of 8 subpopulations between responders, primary resistance and acquired resistance groups within CD4 or CD8 T cells (data not shown).

We also analyzed expression of markers recently reported to correlate with either anti-PD-1 responsiveness (28) or tumor control (34, 35) (Supplementary Figure 3). There was no significant difference in expression of Ki-67, TOX, or TCF1/TCF7, either between the three treatment groups or over time within each group (data not shown).

### Expression of Inhibitory Receptors on CD8 T Cells According to Their Differentiation Status Over the Course of Treatment

**Figure 5:**
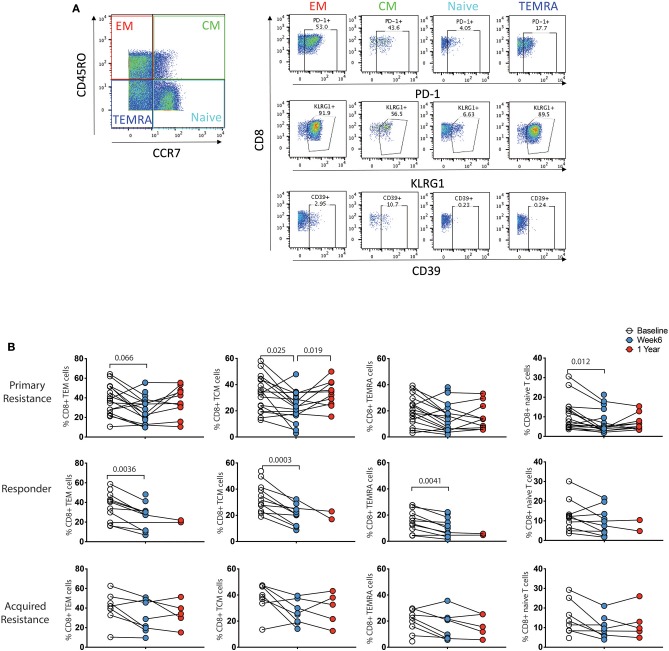
Differential expression pattern of PD-1 within effector/memory compartments of CD8 T cells. **(A)** Gating strategy to distinguish effector memory (TEM), central memory (TCM), effector memory CD45RA (TEMRA), and naïve compartments. **(B)** Longitudinal analysis of PD-1 expression. Baseline, open circles; week 6, blue filled circles; 1 year, red filled circles. Differences in frequencies of immune cell populations between the 3 timepoints were assessed using a repeated measures mixed effects analysis followed by Tukey's multiple comparisons test and *p*-values are indicated.

Previous studies have shown differences in exhaustion markers according to the differentiation status of T cells (21). As shown in Figure 5A, four subpopulations of CD8 T cells were defined by expression of CCR7 and CD45RO: naïve (CCR7^+^CD45RO^−^), central memory (TCM, CCR7^+^CD45RO^+^), effector memory (TEM, CCR7^−^CD45RO^+^) and terminally differentiated effector memory (TEMRA, CCR7^−^CD45RO^−^) (36). We did not observe significant associations between these T cell subgroups and clinical response to immunotherapy at baseline or week 6 time points (data not shown). To test whether the CD8 T cell subpopulations showed differential reduction in PD-1 expression during therapy, we compared longitudinal patterns of expression (Figure 5B). PD-1 expression at baseline was highest in the TEM and TCM subpopulations in all 3 patient groups. There were drops in PD-1 expression within all 4 CD8 T cell subpopulations in the responder and primary resistance patient groups, some of which reached statistical significance, indicating that the reduction shown in Figure 4A was broadly present in all CD8 T cells.

### The Transcription Factor Eomes in CD8 T Cells Associates With High Levels of Inhibitory Receptors and Cell Division Whereas T-Bet Associates With Cytotoxic Effector Molecules

**Figure 6:**
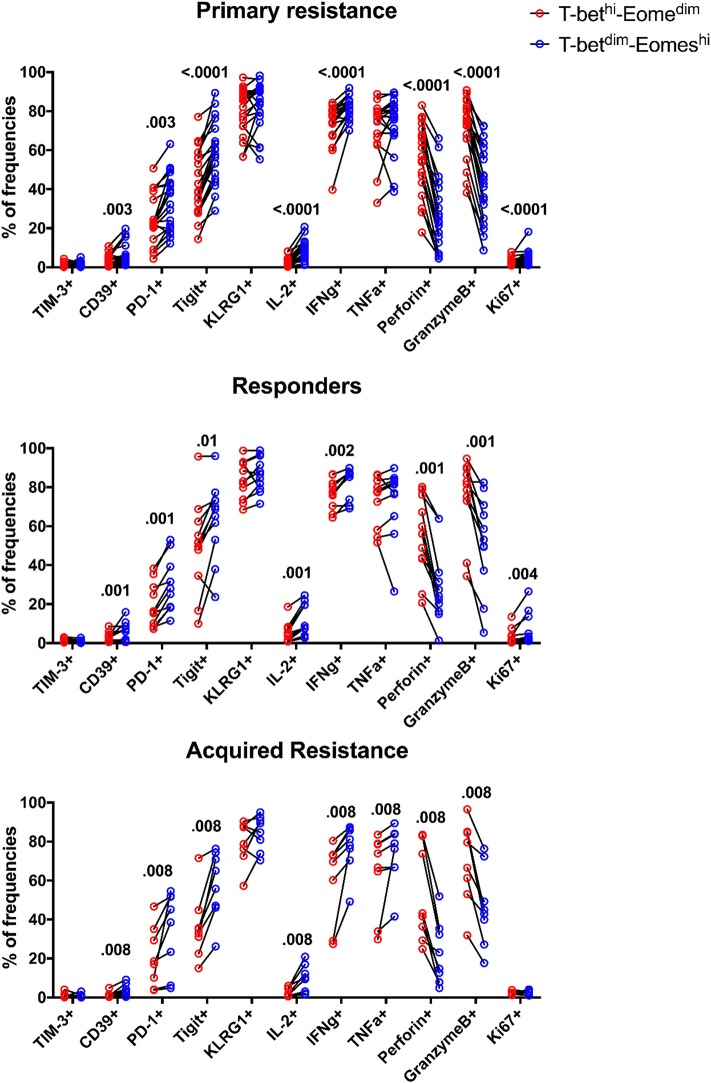
Expression patterns of inhibitory receptors and cytokines on different subsets of CD8 T cells. The frequency of TIM-3, CD39, PD-1, TIGIT, KLRG1, IL-2, IFNγ, TNFα, Perforin, GranzymeB, and Ki-67 expressing cells within total T-bet^dim^Eomes^hi^ (blue) and T-bet^hi^Eomes^dim^ (red) CD8 T cells for all baseline samples. Statistical analysis was performed with a Wilcoxon matched-pairs single rank test.

A number of transcription factors (TFs) are known to be involved in the differentiation of T cells and to undergo changes associated with the development of T cell exhaustion. The TF T-bet was reported to regulate Th1 responses of CD4, CD8 T cells, and innate cells (37) whereas the TF Eomesodermin (Eomes) has been shown to be required to induce production of effector molecules such as granzyme B and perforin (38). In view of this, we studied expression of T-bet and Eomes in the 3 groups of patients (Supplementary Figure 5) and their impact on effector molecules and exhaustion markers (Figure 6). We did not observe any significant differences in expression of T-bet, Eomes, T-bet^dim^/Eomes^hi^, or T-bet^hi^/Eomes^dim^ populations between the three subject groups (Supplementary Figure 5). Furthermore, no alteration in the ratio of T-bet to Eomes expression was observed in any of the groups (Supplementary Figure 5C).

The expression of Eomes, however, was linked to expression of the inhibitory receptors TIGIT, KLRG1, PD-1, and CD39 in that their expression was associated with high levels of Eomes and low levels of T-bet (Figure 6). This also applied to Ki-67, a marker of cell division, and production of IL-2 and IFNγ. In contrast, the effector molecules perforin and granzyme B were associated with high levels of T-bet and low levels of Eomes. Taken together, these results indicate that an inverse expression pattern of T-bet and Eomes is highly associated with the up-regulation of several inhibitory receptors and cytokines as well as Ki-67 for total CD8 T cells, independently of anti-PD-1 therapy outcome in melanoma patients.

### Analysis of Cytokine Production by CD4 and CD8 T Cells During Immunotherapy

**Figure 7:**
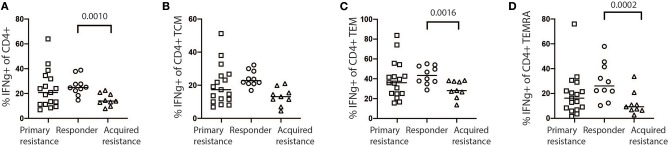
Expression of IFNγ by stimulated baseline samples. **(A)** Comparison of primary resistance, responder, and acquired resistance groups revealed production by a significantly higher proportion of total CD4 T cells within the responder group, compared with the acquired resistance group, however not significant within the CD4 T cell CM compartments **(B)**. The difference was also statistically significant within the CD4 T cell EM **(C)** and TEMRA **(D)** compartments. The statistical differences were analyzed using a Kruskall-Wallis multiple comparisons test.

Exhausted T cells have been reported to have reduced production of cytokines such as IL-2 and IFNγ and reduced expression of the effector molecules perforin and granzyme B (18). We examined CD4 and CD8 T cells for expression of granzyme B, IFNγ, IL-2, Ki-67, perforin, and TNFα as shown in Supplementary Figure 6A. In general, we observed higher levels of granzyme B, IFNγ, IL-2, and perforin expression by CD8 T cells compared to CD4 T cells (Supplementary Figure 6B). No significant differences in expression of granzyme B, IL-2, Ki-67, perforin, and TNFα by CD8 and CD4 T cells were observed between the treatment groups at any time point (Supplementary Figure 6B), with the exception of increased IFNγ production by CD4 T cells in baseline samples from the responder group compared with the acquired resistance group (Figure 7). In addition, we compared IFNγ production by NK cells and γδT cells and found no significant differences between the groups at any timepoint.

### Dimensionality Reduction and Visualization

Because manual gating is only rarely applied to mass cytometry data, we also applied t-SNE analysis to our dataset to check whether additional differences between response groups would become apparent (Supplementary Figures 7–10). For unstimulated samples, tSNE indicated a trend toward fewer NK cells and more myeloid cells (principally CD14^+^ classical monocytes) in the primary resistance group compared to the responder group, with the acquired resistance group intermediate between the two (Supplementary Figure 7). Visualization of marker expression (Supplementary Figure 8) did not reveal any obvious differences between the 3 groups. The distribution of markers was consistent with the normal expression patterns that had already been revealed by our manual gating. For the stimulated samples, there was trend toward higher IFNγ expression by CD4 T cells in the responder group, consistent with our manual gating results (Supplementary Figures 9, 10).

## Discussion

A number of studies have suggested that the development of the T cell exhaustion state may limit the effectiveness of the anti-tumor immune response. Studies on T cells infiltrating melanoma have shown that T cell exhaustion states were relatively common (25) and associated with low response rates (26). It was unknown, however, whether such states could be identified in circulating PBMCs, and whether they had the potential to serve as a more convenient measure in management of immunotherapy. This prompted the present study on a cohort of patients who had been treated by ICB and who had the expected variation in response to treatment. The study had access to highly sensitive multiparameter detection facilities. The resulting data were analyzed using expert manual gating.

Our results show that examination of different mononuclear populations reveals relatively few differences between patients who responded to treatment compared to non-responders or those failing treatment within 1 year of start of therapy. These differences were largely evident in pre-treatment samples and carried through at the 6 week and 1 year follow up timepoints. Responders had a higher frequency of NK and CD56-expressing T cells, while patients in the primary resistance group had a higher frequency of classical monocytes. Visualization of the same data using t-SNE did not reveal any additional differences between the patient groups. A number of previous publications have reported that high circulating monocyte numbers are associated with a worse prognosis (39, 40), although a recent study of PBMCs from melanoma patients reported the opposite finding in patients treated with ICB (29). As in the latter study, we saw no significant differences in numbers of total CD4 or CD8 T cells, or in CD4 or CD8 T cell naïve and memory cell subsets between the groups prior to or during treatment. The responder group had higher IFNγ production in CD4 T cells at baseline, compared with the acquired resistance group. The exhaustion markers CD39, PD-1, TIGIT, TIM-3, and KLRG1 were detected on CD8 and CD4 T cells although TIM-3 was at low levels, consistent with so called progenitor exhausted T cells described by others (24). There were no significant differences in expression of exhaustion markers between the groups prior to treatment. At the 6 week timepoint, PD-1 was downregulated, as expected, and CD39 expression was increased in responder and primary resistance groups. Low patient numbers in the acquired resistance group limited the significance of differences seen at week 6.

It was reasoned that exhaustion markers may be expressed by relatively few T cells recirculating from the tumor, and may be restricted to certain differentiation subsets such as effector memory subsets. Variability of expression of exhaustion markers according to differentiation of T cells is well-described by others (21). In a study on TILs, exhaustion markers were found on central memory cells expressing PD-1 and the transcription factor Tcf1 (34). These cells mediated proliferative responses to immunotherapy and were detected in the blood of melanoma patients briefly in the first week after treatment. Single cell studies in TILs from melanoma patients also found that the dysfunctional T cells were the major proliferative subset, constituted up to ~70% of the TILs, and were separate to cytotoxic subsets (25). Another single cell analysis including mass cytometry of exhausted T cells from patients with viral infections or lung cancers described up to 9 different T cell exhaustion states that could be modified by therapy (21, 41).

In view of these studies we examined the expression of exhaustion markers on naïve, effector memory, central memory and terminally exhausted subsets. As expected, there were differences in expression of the markers in these subsets but importantly these did not differ between the patient groups at baseline or at the 6 week and 1 year treatment timepoints. The question remains whether particular subsets of exhausted cells as described in other diseases such as lung cancer, HIV or other infections may have differed between the subgroups (41) but a wider range interrogation would be needed to examine this.

Previous studies by Sen et al. (20) and Pauken et al. (22) have shown that the differentiation state referred to as T cell exhaustion is associated with epigenetic changes that allow interaction with transcription factors that regulate the differentiation state. Tcf1 appears to be essential in sustaining exhausted T cells and to be associated with expression of PD-1 and LAG-3 (42). A single cell analysis on TILs from 48 melanoma samples also implicated Tcf7 in positive outcomes to therapy (26). In peripheral blood, we found no difference in expression of Tcf7 in patients who did or did not respond to anti-PD-1 therapy. Eomes was identified together with other TFs as being associated with CD8 T cell dysfunction in the study by Li et al. (25). Our study on TFs implicated Eomes in T cell exhaustion in that high Eomes was associated with expression of TIGIT, PD-1, KLRG1, CD39, and Ki-67 whereas the effector molecules perforin and granzyme B were reduced. The low levels of IL-2 and reduced levels of TNFα and IFNγ are consistent with the cytokine levels shown in CD8 T cells. We reasoned that responses to ICB may be associated with T-bet driving Th1 type responses. There was a significant increase in CD4 IFNγ responses in the responders compared to the acquired resistance group, but not the primary resistance group. The reasons for this difference are not known. We examined whether the ratio of T-bet to Eomes in pre-treatment and 6 week samples would be a more sensitive measure of responses, but no differences were seen between the 3 groups. This indicated that high Eomes or T-bet:Eomes ratios could not be used as a guide to responses to ICB treatment. Eomes has been identified by others as being associated with CD8 T cell dysfunction (25). In addition, T-bet^high^Eomes^low^ CD8 T cells have been identified as an intermediate exhausted CD8^+^ T cell population that could be reinvigorated by checkpoint inhibitors (43, 44). Further work to understand the disparate roles of T-bet and Eomes in regulating T cell function is needed.

The association of the major CD16^hi^CD56^+^ subset of NK cells with responses to ICB treatment requires further analysis. NK cells are known to express PD-1 and other checkpoint receptors (45) and their cytotoxic activity *in vitro* is increased by PD-1 blockade (46). A study on 25 melanoma patients with metastatic disease reported that patients with higher numbers of intra- and peri-tumoral NK cells had higher response rates than those with lower NK cells (47). Previous studies on 40 patients treated with anti-PD-1 also found that responders had higher numbers of CD56^+^CD16^+^CD69^+^ NK cells in pretreatment blood samples after activation *in vitro* (30). That study also found no differences in CD4 or CD8 memory T cell subsets between responders vs. non-responders. These results need to be considered together with the inverse correlation shown between survival and CD56^bright^CD16^lo^ NK cells in blood of 29 patients with stage III/IV melanoma (48). There is also a long history of relatively low rates of response to adoptive treatment with IL-2 activated lymphokine activated killer cells that included NK cells (49). It remains possible that the CD56^bright^CD16^lo^ NK cells have an immunoregulatory role that indirectly increases adaptive responses by CD8 T cells, as described by others (50–52).

A subset of CD3 positive cells expressing CD56 was also assessed in the current study. CD56 expression on T cells is known to be associated with potent effector function in human peripheral blood (53). CD56^+^ T cells were present at higher frequency in responders compared to the primary resistance group, and the differences between individuals were maintained throughout ICB therapy.

In conclusion, the intent of these studies was to identify biomarkers in blood that would be readily accessible and useful in planning treatments that may reduce T cell exhaustion states, such as treatment with epigenetic regulators (54). Although they were comprehensive in including a large number of markers associated with T cell exhaustion that had previously been validated by studies on TILs, the panel of exhaustion markers selected for this study did not distinguish patients who responded from those who did not respond to anti-PD-1 treatments. Evolving studies in other cancers and viral infections point to a number of different states of exhaustion/dysfunction that undergo dynamic changes that may not have been captured by the procedures used in this study (41). For example, it is possible that entry into the immunosuppressive tumor microenvironment may induce changes in tumor-specific T cells. In addition, the number of “bystander” T cells in blood may have also made detection of relevant small tumor-specific populations difficult and study of clonally restricted T cells that are also seen in the tumor may have identified a population more relevant to clinical outcomes, but such studies were beyond the scope of this study. Rather similar conclusions were reached in monitoring responses to hepatitis C infections where patients clearing infection had similar high PD-1 levels to those not clearing the infection (55, 56). Pre-treatment levels of NK and CD56^+^ T cells and CD4 T cell IFNγ production did however appear to distinguish responders from non-responders and warrant further study.

## Data Availability Statement

The datasets generated for this study are available on request to the corresponding authors.

## Ethics Statement

Patient samples analyzed in the current study were obtained from The Melanoma Institute Australia biospecimen bank with written informed patient consent and institutional review board approval (Sydney Local Health District Human Research Ethics Committee, Protocol No. X15–0454 and HREC/11/RPAH/444).

## Author Contributions

MP, HM, BF, and PH conceived the study and served as the primary authors of the paper. MP, HM, AE, H-YT, JT, BF, and PH planned, performed, and analyzed the majority of the studies, prepared the manuscript, figures, and tables. JL, MC, AM, GL, RS, BF, HM, and PH conducted the relevant clinical trials and provided guidance clinical information and manuscript review. All authors read and approved the final manuscript.

### Conflict of Interest

JL has received travel support from BMS, BioRad and has received honoraria from AstraZeneca. MC is a consultant advisor for BMS, MSD, Novartis, Roche, Ideaya, Sanofi, Merck Serono, Amgen, Nektar and has received honoraria from BMS, MSD and Novartis. AM is on Advisory boards for BMS, MSD, Novartis, Roche and Pierre-Fabre. GL is consultant advisor to Aduro, Amgen, Array, BMS, MSD, Novartis, Pierre-Fabre, Roche. RS has received fees for professional services from Merck Sharp & Dohme, GlaxoSmithKline Australia, Bristol-Myers Squibb, Novartis Pharmaceuticals Australia Pty Ltd, Myriad, NeraCare and Amgen. The remaining authors declare that the research was conducted in the absence of any commercial or financial relationships that could be construed as a potential conflict of interest.
