# Supplementary material for: Pretreatment Innate Cell Populations and CD4 T Cells in Blood Are Associated With Response to Immune Checkpoint Blockade in Melanoma Patients
Source: Front Immunol. 2020 Mar 10;11:372. doi: 10.3389/fimmu.2020.00372 (PMC7076153; doi:10.3389/fimmu.2020.00372)
Supplement: Supplementary file 1 [file Data_Sheet_1.pdf]

Supplementary Material

Supplementary table 1. Characteristics of study participants.

Variable	Responders	Primary resistance	Acquired resistance
Number of patients	13	19	10
Number of samples collected at baseline	10	17	9
Number of samples collected at week6	11	19	9
Number of samples collected at 1 year	2	10	6
Treatment	Pembrolizumab=100%<br>Nivolumab=0%	Pembrolizumab=69%<br>Nivolumab=31%	Pembrolizumab=80%<br>Nivolumab=20%
Median age	77(44-91)	69(41-88)	77(63-86)
Sex			
Male	7(54%)	16(84%)	8(80%)
Female	6(46%)	3(16%)	2(20%)
Mutation			
WT	7(54%)	7(37%)	4(40%)
NRAS	5(38%)	6(32%)	4(40%)
BRAF	0(0%)	4(21%)	2(20%)
KRAS	0(0%)	1(5%)	0(0%)
KIT	1(8%)	1(5%)	0(0%)
M Staging at inclusion			
M1a	0(0%)	1(5%)	2(20%)
M1b	5(38%)	2(11%)	5(50%)
M1c	5(38%)	9(47%)	0(0%)
M1d	1(8%)	5(26%)	0(0%)
IIIc	2(15%)	2(11%)	3(30%)
LDH at inclusion			
Normal	11(85%)	14(74%)	10(100%)
Elevated	2(15%)	5(26%)	0(0%)

Supplementary table 2. The antibody panel used for mass cytometry phenotypic and functional analysis of immune cells in melanoma patients.

Metal label	Specificity	Antibody clone	Manufacturer
Barcoding and live/dead			
194Pt	Dead cells	Cisplatin	
104Pd or 108Pd	CD45	HI30	BD
Surface stain			
140Ce	Beads	n/a	
89Y	CD8A	RPA-T8	Biolegend
113In	CD56	REA196	Miltenyi Biotec
142Nd	CD19	HIB19	BD
143Nd	CD45RA	HI100	BD
144Nd	TCRgd	B1	BD
145Nd	CD4	RPA-T4	BD
148Di	CD5	HI10a	Biolegend
149Sm	TIM-3	7D3	BD
150Nd	KLRG1	SA231A2	Biolegend
151Eu	CD39	A1	Biolegend
152Gd	CD45RO	UCHL1	BD
154Gd	CD3	UCHT1	BD
155Gd	CXCR5	RF8B2	BD
156Gd	PD-1	EH12.2H7	Biolegend
159Tb	CCR7	150503	R&D Systems
161Di	CD274	MIH1	BD
163Dy	CXCR3	REA232	Miltenyi Biotec
164Er	CTLA-4	14D3	eBioscience
165Di	CD16	3G8	BD
166Er	TIGIT	MBSA43	eBioscience
167Di	CD27	M-T271	Biolegend
169Tm	CD25	M-A251	Biolegend
172Yb	CD38	HIT2	BD
173Yb	CD14	M5E2	BD
174Yb	HLA-DR	L243	BD
176Lu	CD127	A019D5	Biolegend
Intracellular stain			
141Pr	TNFα	Mab11	Biolegend
146Nd	Eomes	WD1928	Life Technologies
153Eu	TOX	TXRX10	eBioscience
158Gd	IL-2	MQ1-17H12	BD
160Gd	BCL-2	BCL-2-100	Life Technologies
162Er	FoxP3	PCH101	eBioscience
168Er	IFNγ	B27	BD
170Er	TCF1/TCF7	C63D9	Cell Signalling Technology
171Yb	Granzyme B	GB11	Acris
175Lu	Perforin	dG9	Biolegend
209Bi	T-bet	4B10	BD
115Di	Ki67	B56	BD

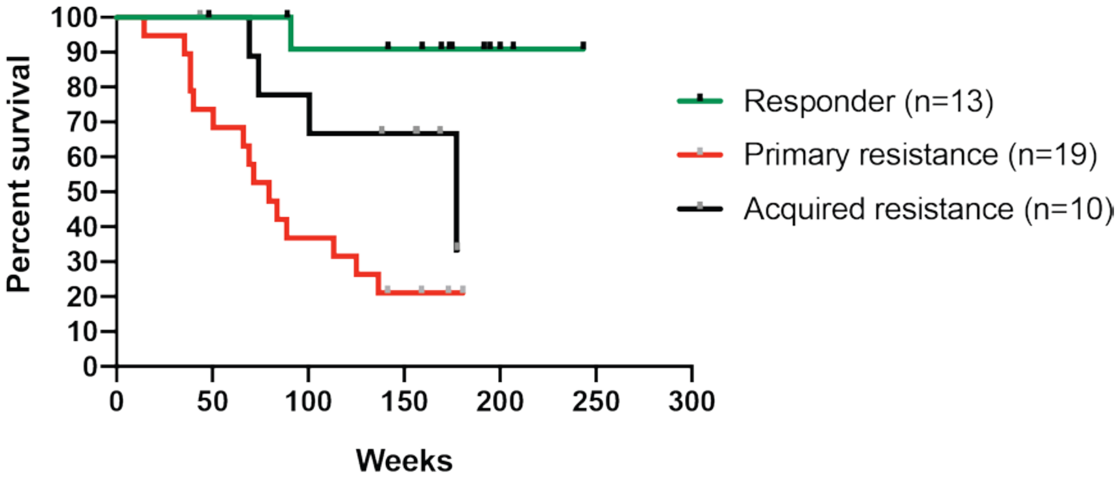

Supplementary Figure 1. Overall survival of melanoma patients categorized as responder vs primary and acquired resistance group. A Kaplan-Meier (KM) plot was generated comparing individual groups. Survival duration was calculated from the first treatment date to the last follow-up date. The median survival of primary resistance and acquired resistance group was 80 and 177 weeks respectively, and the median survival of the responder group was not reached as >50% of patients survived throughout the study period. Statistical analysis performed by the Mantel-Cox test gave a logrank p value of 0.0002.

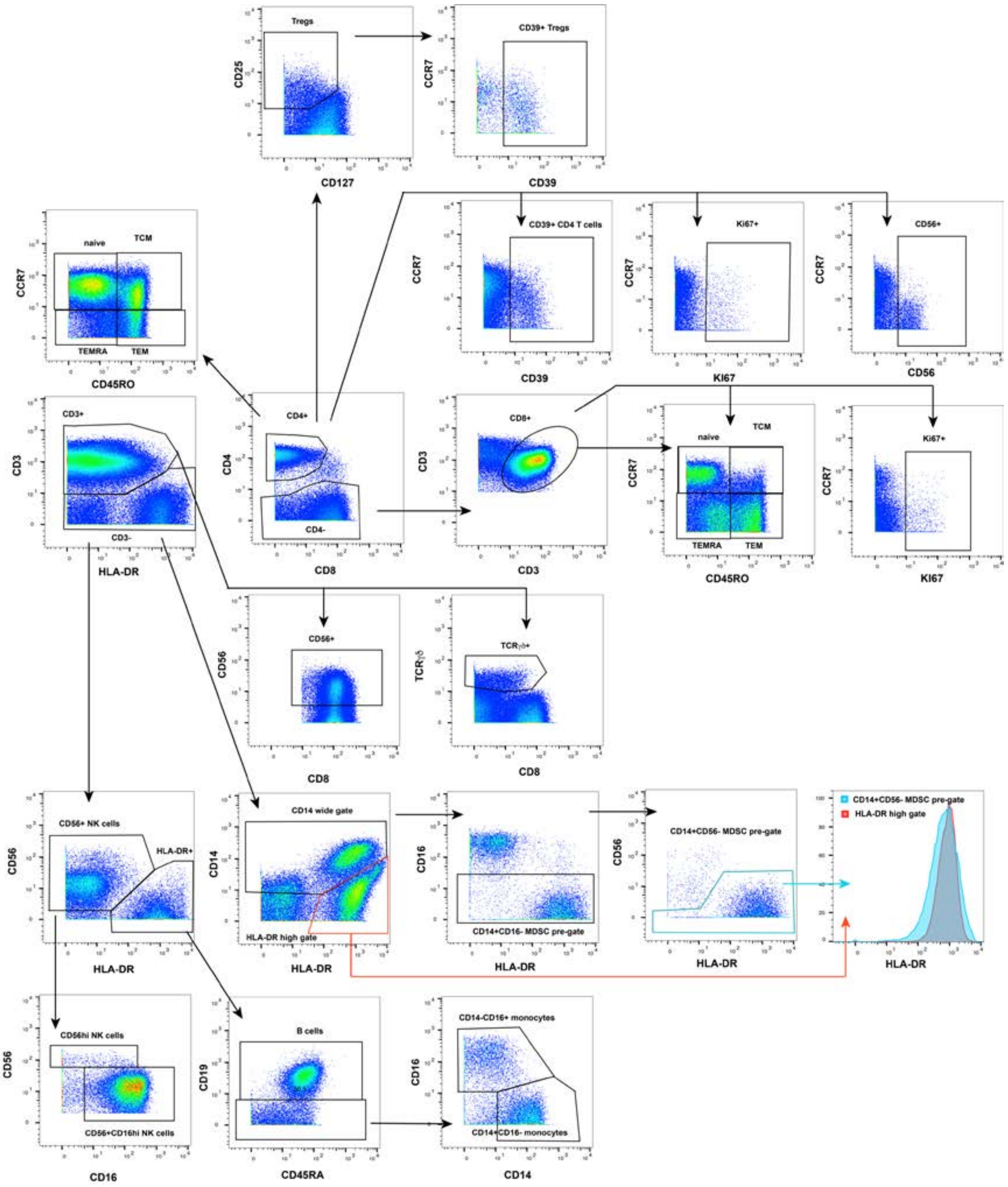

Supplementary Figure 2. Gating strategy for mass cytometry data. Prior to population analysis, cells were pre-gated as positive for DNA-intercalated events (detected on channel 191) and negative for EQ-beads (detected in channel 140), and with subsequent discrimination for exclusion of dead (cisplatin positive) cells and differential CD45 antibody staining to distinguish barcoded, concurrently analysed samples. Dotplots show a representative example of the gating strategy used to identify multiple lymphocyte lineages and subpopulations. For MDSC analysis, expression of HLA-DR by CD14$^+$ cells was compared with expression of HLA-DR by B cells. No HLA-DR-negative population was apparent and the magnitude of the left shift of CD14 vs B cells was not significantly different between any of the patient groups.

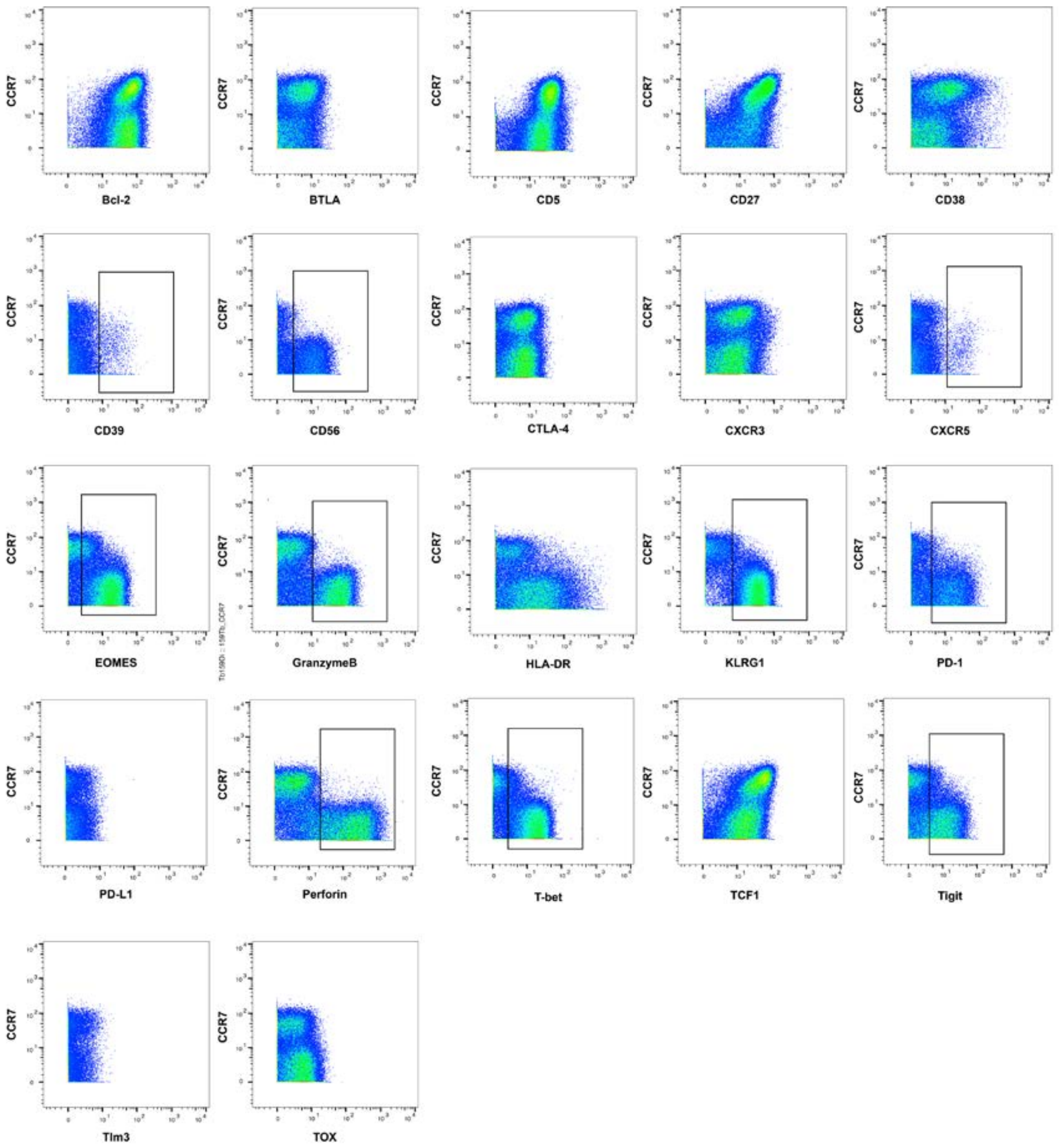

Supplementary Figure 3. Representative marker expression by CD8 T cells in the absence of stimulation. CD39, CD56 CXCR5, Eomes, GranzymeB, KLRG1, PD-1, Perforin, T-bet and Tigit were predominantly expressed by CCR7- cells and were readily gated, as were Ki67 and the CCR7/CD45RO populations shown in Supplementary Figure 2. Mean expression of Bcl-2, CD5, CD27, CD38, CXCR3, and TCF1 correlated with CCR7 but was not amenable to conventional gating. HLA-DR and TOX were negatively correlated with CCR7.

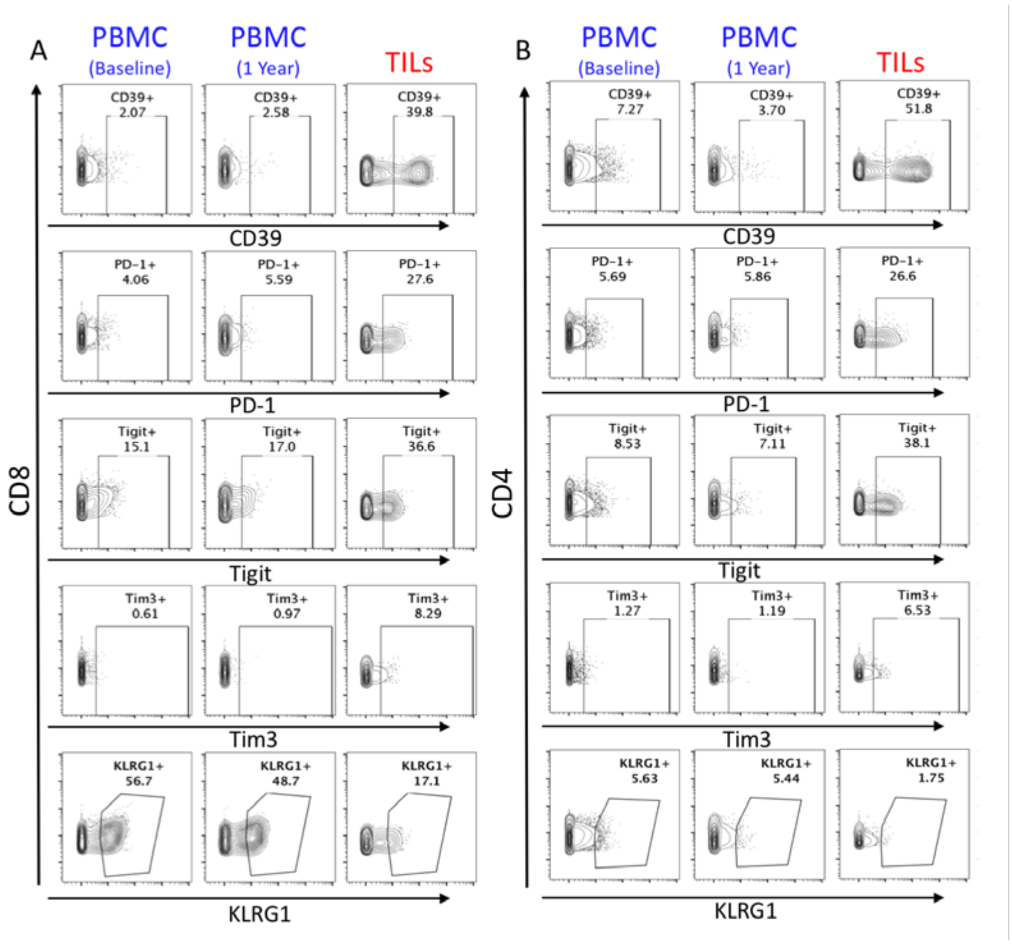

Supplementary Figure 4. TILs from metastatic melanoma tumor dissociates predominantly express checkpoint molecules CD39, TIGIT and PD-1 compared to PBMCs. (A) CD8 T cells and (B) CD4 T cells from a paired PBMC and TILs (13-month post treatment) were assessed for expression of various checkpoint molecules by CyTOF.

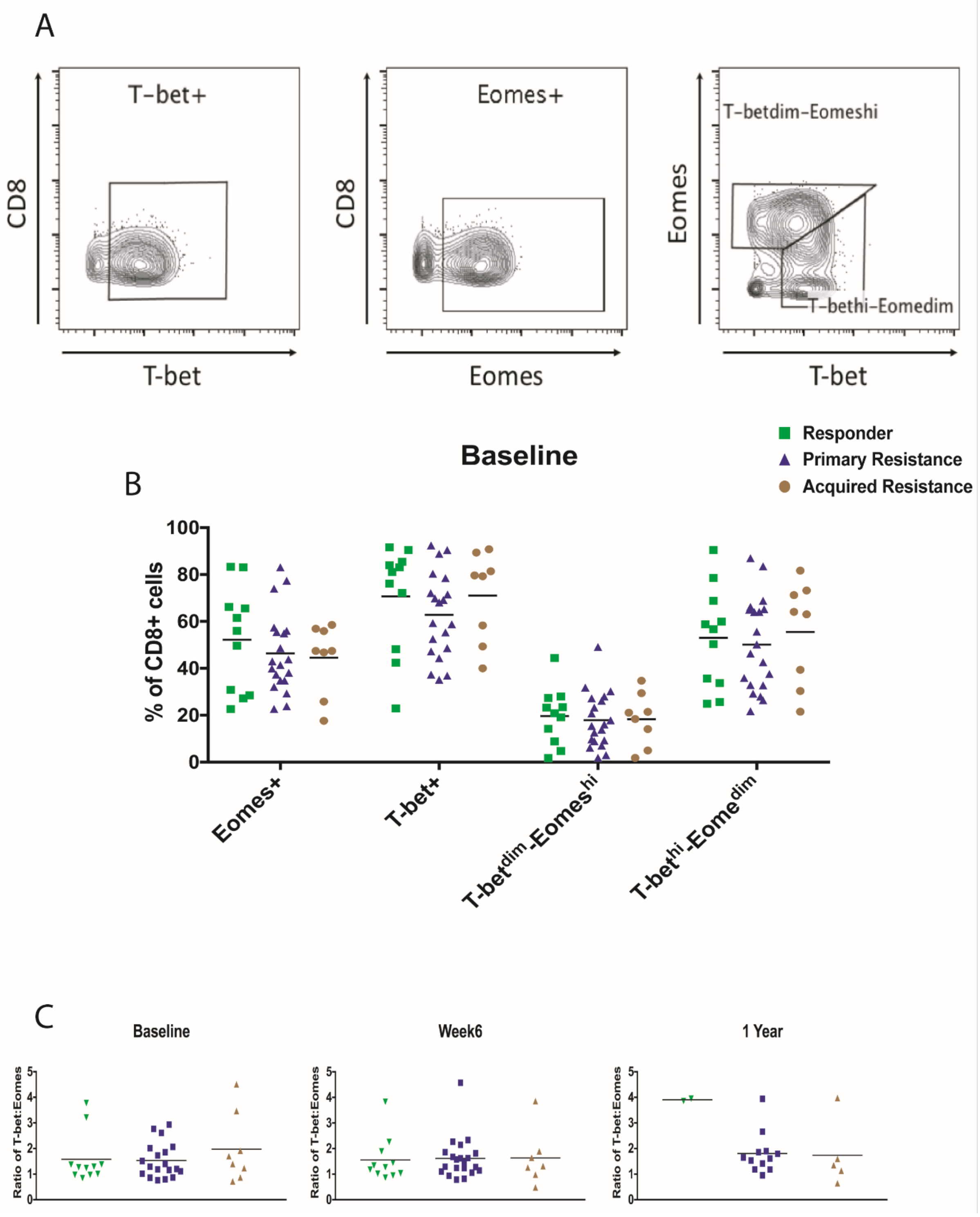

Supplementary Figure 5. Expression patterns of T-bet and Eomes within CD8 T cells. (A) Gating strategy to distinguish the T-bet and Eomes populations. (B) The percentage expression of T-bet and Eomes and co-expression of T-bet^{dim}Eomes^{hi} and T-bet^{hi}Eomes^{dim} on total CD8 T cells in melanoma patients prior to anti-PD-1 treatment. (C) Data showing the T-bet^{+}: Eomes^{+} CD+ T cells ratio in responders (green) versus primary resistance (blue) and acquired resistance (brown).

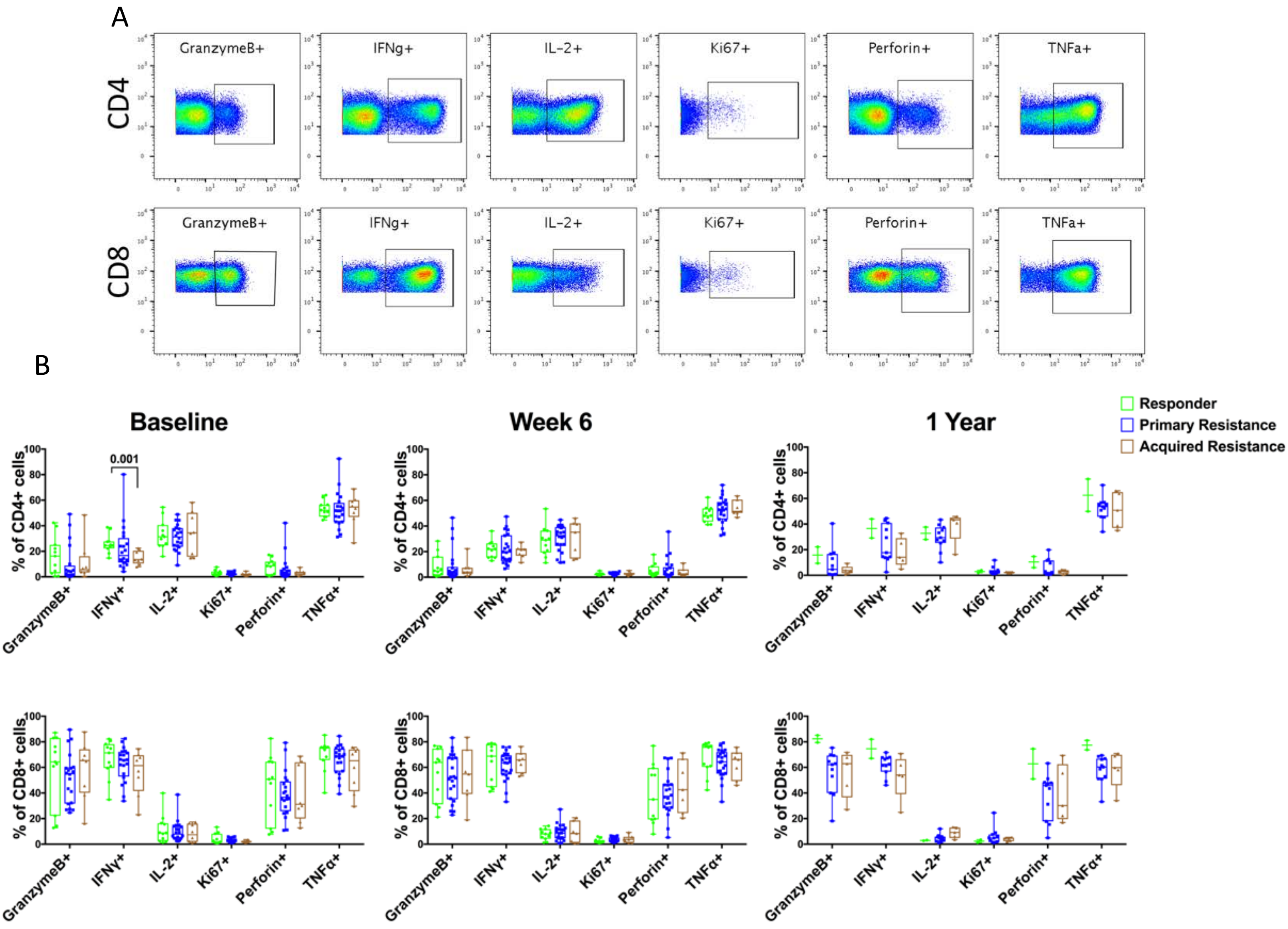

Supplementary Figure 6. Expression of cytotoxic effector proteins and cytokines in PMA and Ionomycin stimulated CD4 and CD8 T cells from patients with metastatic melanoma. (A) Representative CytoF plots displaying intracellular protein staining for GranzymeB, IFNγ, IL-2, Ki-67, perforin and TNFα within CD4 and CD8 T cells. (B) Comparison of intracellular expression of GranzymeB, IFNγ, IL-2, Ki-67, perforin and TNFα by stimulated CD4 and CD8+ T cells from responder, primary resistance and acquired resistance groups. Box plots indicate median and upper and lower quartiles; the whiskers indicate the 5th and 95th percentiles respectively.

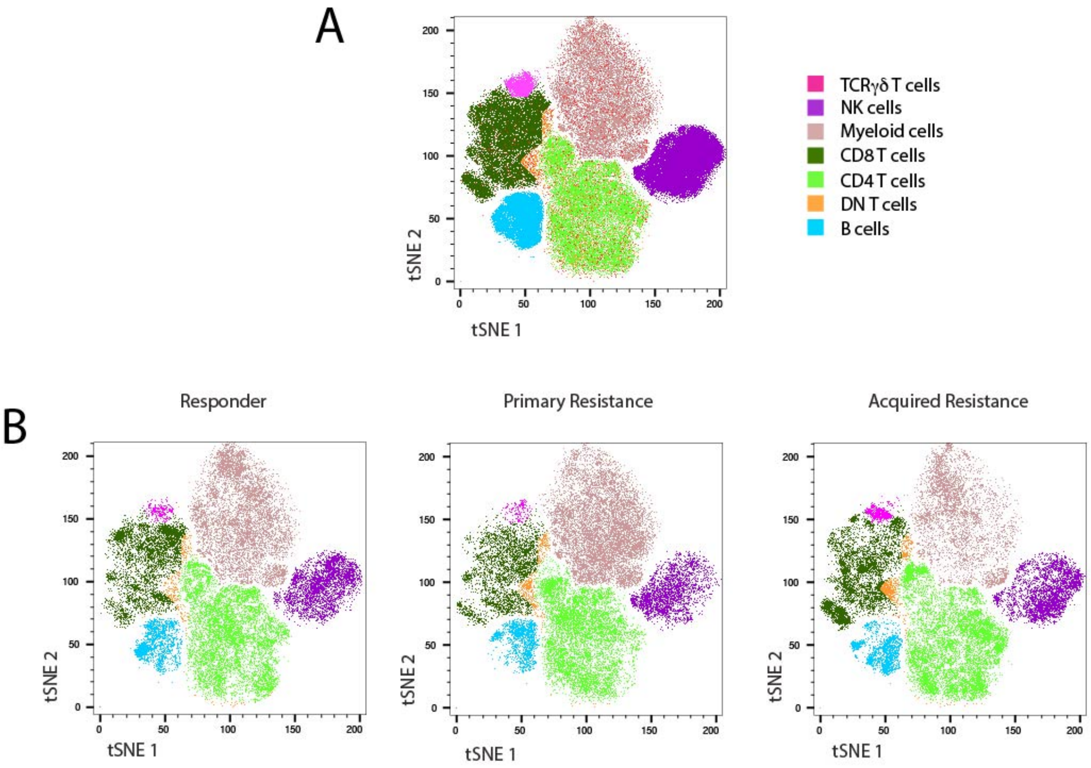

Supplementary Figure 7. t-SNE analysis of mass cytometry data from unstimulated samples. (A) A fixed number of events (1000) from each patient sample plus the batch controls were concatenated into a single file for t-SNE analysis. (B) Individual patient files were then identified within the t-SNE file and pooled into response groups that were downsampled to the minimum present in any group (22,000) so that each visualisation comprised the same number of events.

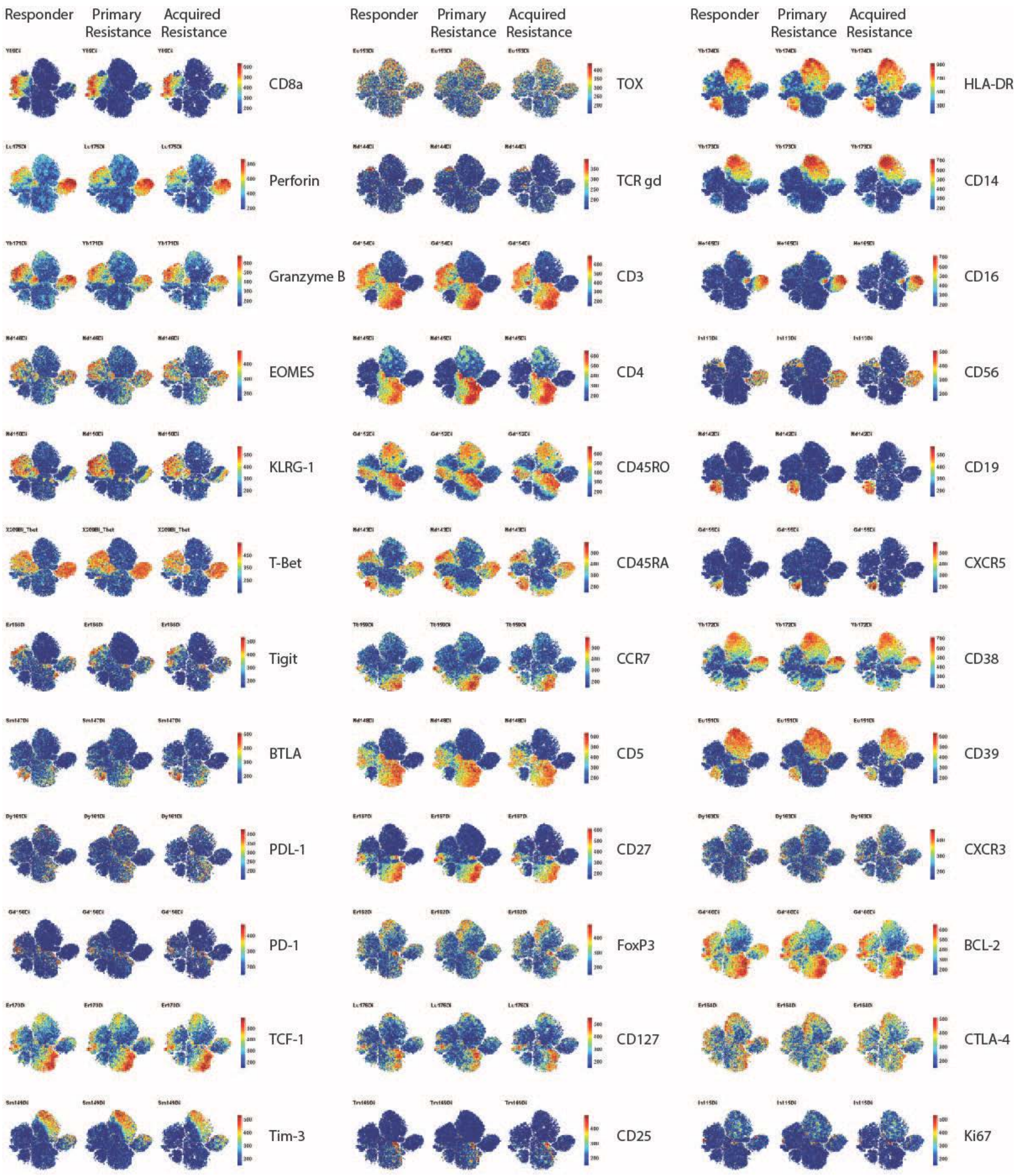

Supplementary Figure 8. Marker expression after t-SNE analysis of mass cytometry data from unstimulated samples. Expression of 36 markers in the pooled response group files shown in Supplementary Figure 7B.

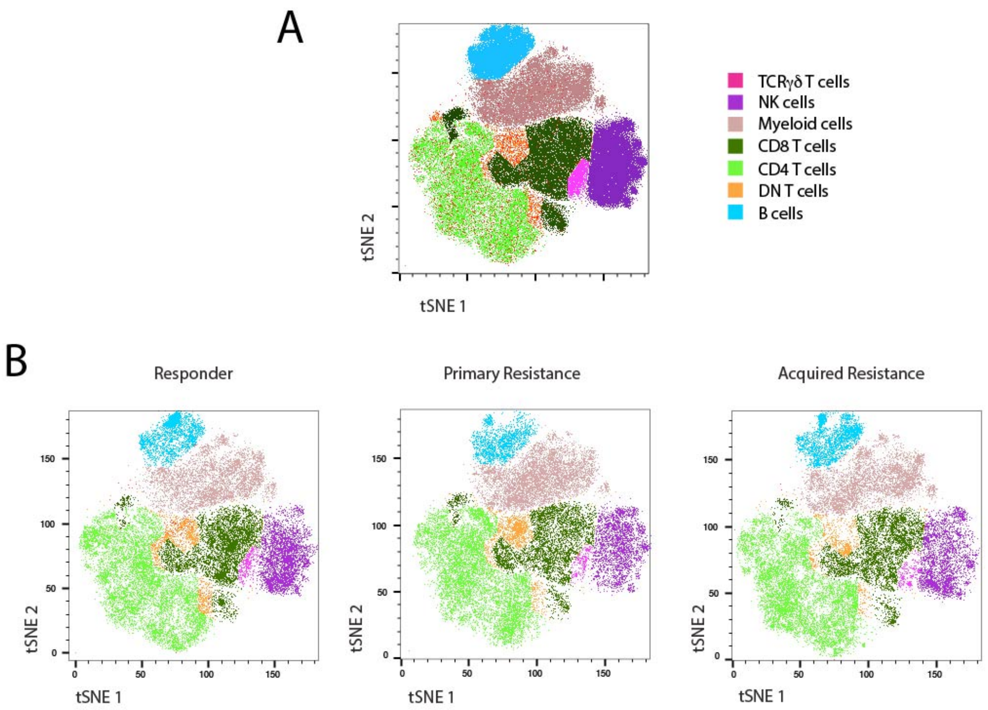

Supplementary Figure 9. t-SNE analysis of mass cytometry data from stimulated samples. (A) A fixed number of events (1000) from each patient sample plus the batch controls were concatenated into a single file for t-SNE analysis. (B) Individual patient files were then identified within the t-SNE file and pooled into response groups that were downsampled to the minimum present in any group (23,000) so that each visualisation comprised the same number of events.

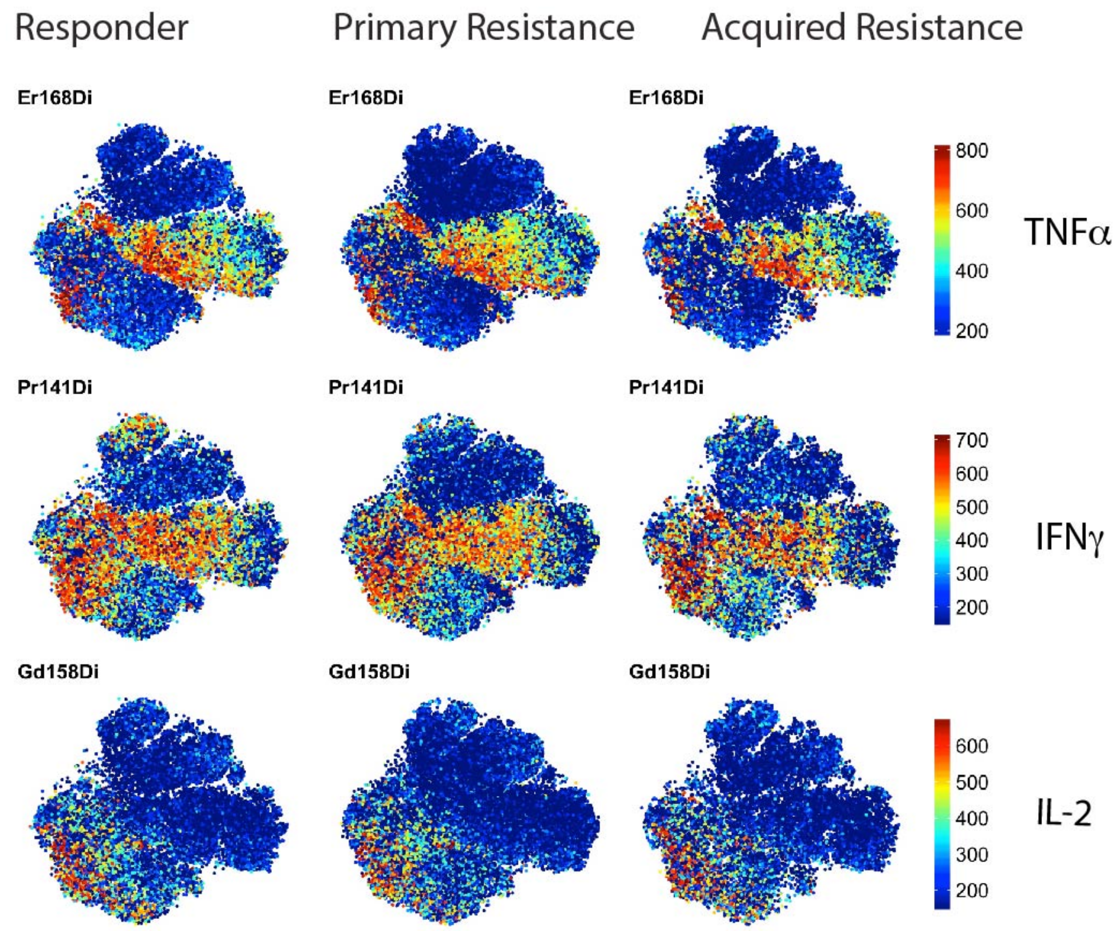

Supplementary Figure 10. Marker expression after t-SNE analysis of mass cytometry data from unstimulated samples. Expression of cytokines in the pooled response group files shown in Supplementary Figure 9.
